# Supplementary figures and images for: Hospitalizations among adults with chronic kidney disease in the United States: A cohort study
Source: PLoS Med. 2020 Dec 11;17(12):e1003470. doi: 10.1371/journal.pmed.1003470 (PMC7732055; doi:10.1371/journal.pmed.1003470)

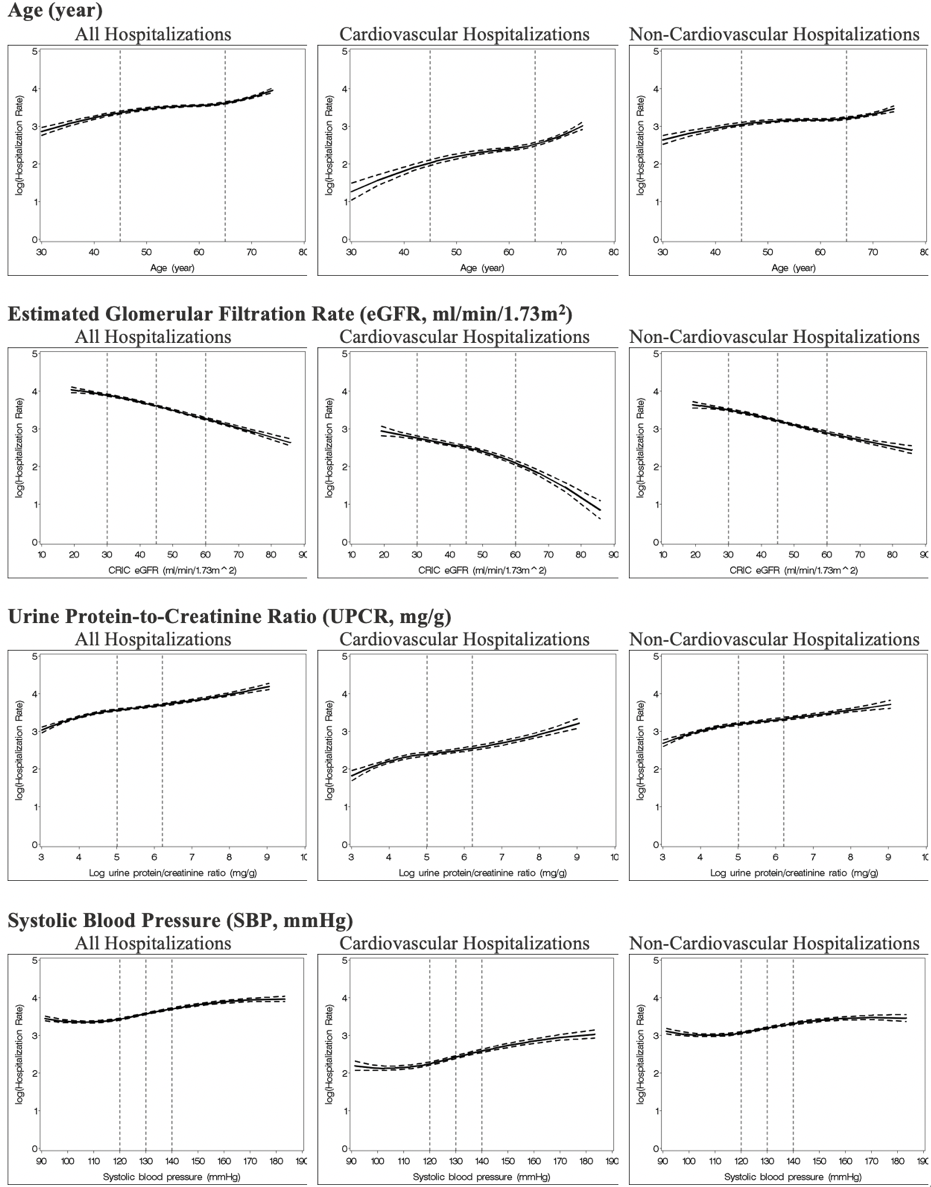

Supplement: S1 Fig — (TIF) [file pmed.1003470.s001.tif]

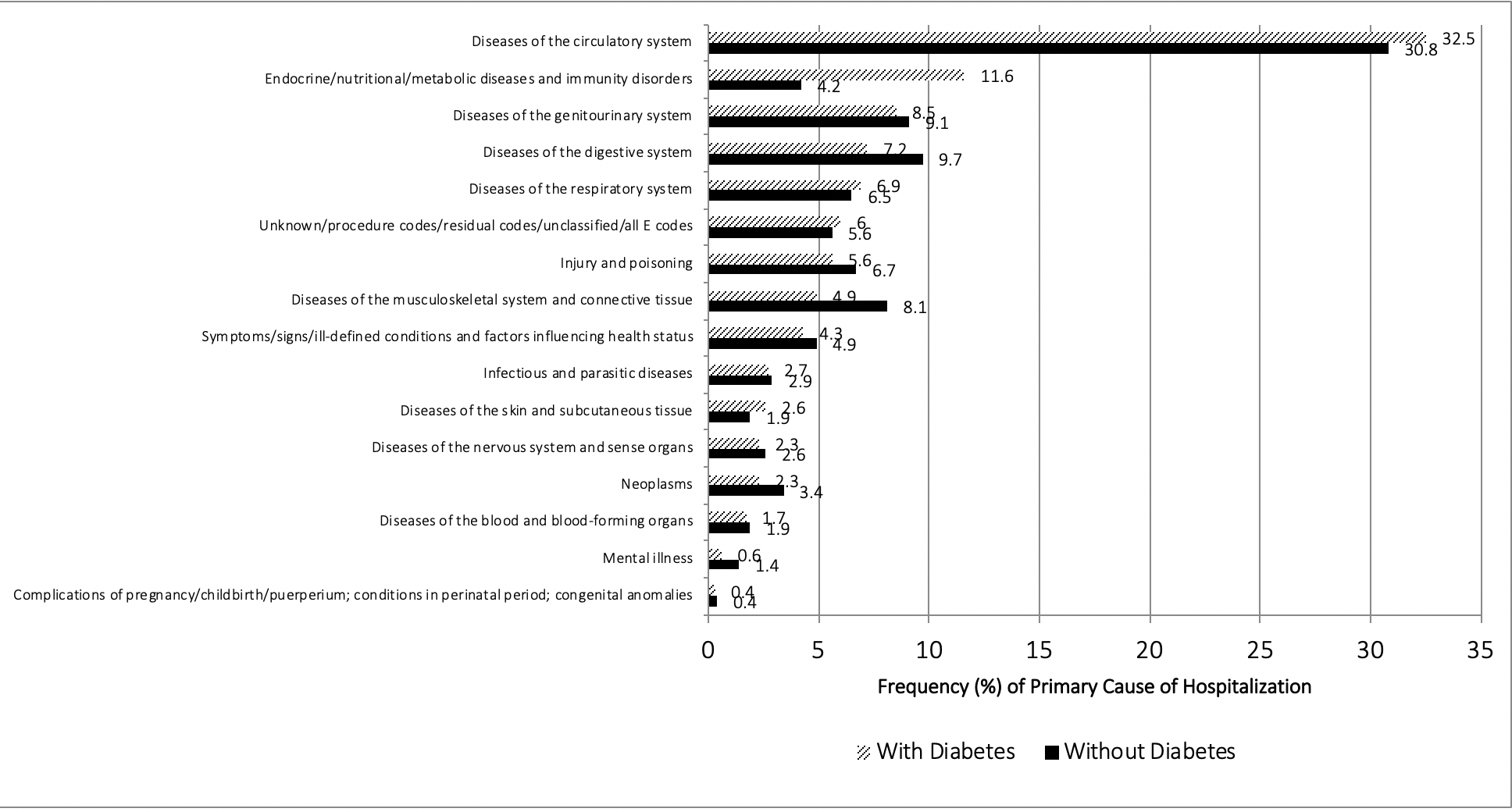

Supplement: S2 Fig — (TIF) [file pmed.1003470.s002.tif]

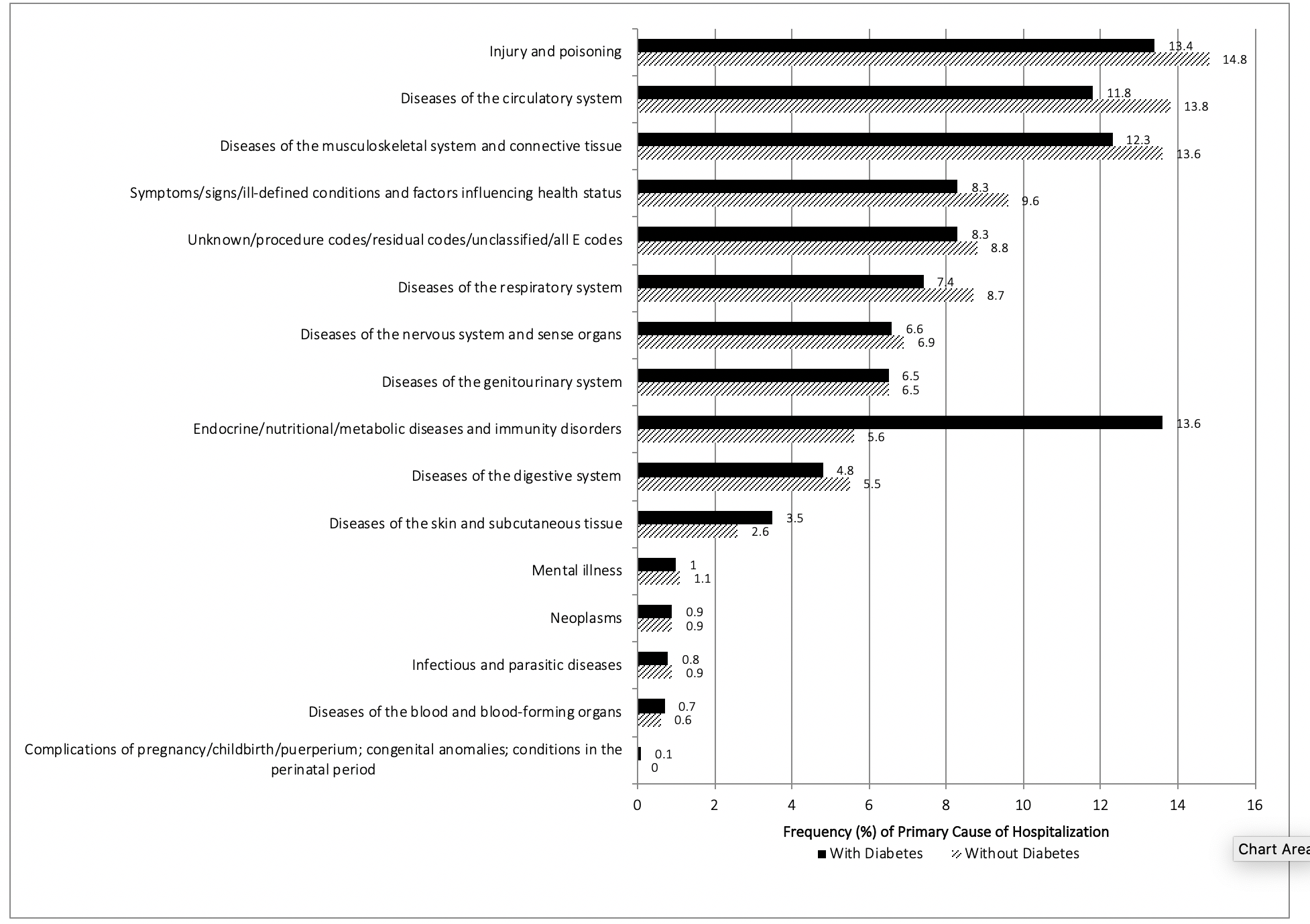

Supplement: S3 Fig — (TIF) [file pmed.1003470.s003.tif]
