# Supplementary material for: Hospitalizations among adults with chronic kidney disease in the United States: A cohort study
Source: PLoS Med. 2020 Dec 11;17(12):e1003470. doi: 10.1371/journal.pmed.1003470 (PMC7732055; doi:10.1371/journal.pmed.1003470)
Supplement: S1 Table — (DOCX) [file pmed.1003470.s004.docx]

**S1 Table: Modified STROBE Statement**

|  | Item No | Recommendation |
| --- | --- | --- |
| **Title and abstract** | 1 | (*a*) Indicate the study’s design with a commonly used term in the title or the abstract  Title; Abstract: methods & Findings |
|  |  | (*b*) Provide in the abstract an informative and balanced summary of what was done and what was found  Abstract: Methods & Findings and Conclusions |
| Introduction | | |
| Background/rationale | 2 | Explain the scientific background and rationale for the investigation being reported  Introduction: paragraphs 1-2 |
| Objectives | 3 | State specific objectives, including any prespecified hypotheses  Introduction: paragraph 3 |
| Methods | | |
| Study design | 4 | Present key elements of study design early in the paper  Found in methods – paragraphs 1 and 2 |
| Setting | 5 | Describe the setting, locations, and relevant dates, including periods of recruitment, exposure, follow-up, and data collection  Found in methods – paragraph 1 |
| Participants | 6 | (*a*) *Cross-sectional study*—Give the eligibility criteria, and the sources and methods of selection of participants  Found in methods – paragraphs 1 and 2 |
| Variables | 7 | Clearly define all outcomes, exposures, predictors, potential confounders, and effect modifiers. Give diagnostic criteria, if applicable  Found in methods: paragraphs 3 and 4 |
| Data sources/ measurement | 8* | For each variable of interest, give sources of data and details of methods of assessment (measurement).  Found in methods: paragraphs 1 and 2; S1 Text |
| Bias | 9 | Describe any efforts to address potential sources of bias  Methods: paragraph 7 |
| Study size | 10 | Explain how the study size was arrived at (if applicable)  Methods: paragraphs 1 and 2 |
| Quantitative variables | 11 | Explain how quantitative variables were handled in the analyses. If applicable, describe which groupings were chosen and why  Methods: paragraphs 4 and 7 |
| Statistical methods | 12 | (*a*) Describe all statistical methods, including those used to control for confounding |
|  |  | (*b*) Describe any methods used to examine subgroups and interactions |
|  |  | (*c*) Explain how missing data were addressed |
|  |  | (*d*) *Cross-sectional study*—describe analytical methods taking account of sampling strategy |
|  |  | (*e*) Describe any sensitivity analyses  Methods: paragraphs 5-8  S2 Text |
| Results | | |
| Participants | 13* | (a) Report numbers of individuals at each stage of study—eg numbers potentially eligible, examined for eligibility, confirmed eligible, included in the study, completing follow-up, and analyzed |
|  |  | (c) Use of a flow diagram  Results: paragraph 1 |
| Descriptive data | 14* | (a) Give characteristics of study participants (eg demographic, clinical, social) and information on exposures and potential confounders |
|  |  | (b) Indicate number of participants with missing data for each variable of interest |
|  |  | (c) *Cohort study*—Summarise follow-up time (eg, average and total amount)  Found in results – paragraph 1; Table 1 |
| Outcome data | 15* | *Cohort study*—Report numbers of outcome events or summary measures over time |
|  |  | *Case-control study—*Report numbers in each exposure category, or summary measures of exposure |
|  |  | *Cross-sectional study—*Report numbers of outcome events or summary measures  Found in results – paragraph 1 |
| Main results | 16 | (*a*) Give unadjusted estimates and, if applicable, confounder-adjusted estimates and their precision (eg, 95% confidence interval). Make clear which confounders were adjusted for and why they were included  Found in results: paragraphs 2,3,5; Figure 1,2, 4, 5; S2, S3, S4 Tables |
| Other analyses | 17 | Report other analyses done—eg analyses of subgroups and interactions, and sensitivity analyses  Found in results – paragraphs 4, 6; Figure 3; S5 Table; S2 Figure |
| Discussion | | |
| Key results | 18 | Summarise key results with reference to study objectives  Found in discussion – paragraph 1, 2, 9 |
| Limitations | 19 | Discuss limitations of the study, taking into account sources of potential bias or imprecision. Discuss both direction and magnitude of any potential bias  Found in discussion – paragraph 8 |
| Interpretation | 20 | Give a cautious overall interpretation of results considering objectives, limitations, multiplicity of analyses, results from similar studies, and other relevant evidence  Discussion – paragraphs 307 |
| Generalisability | 21 | Discuss the generalisability (external validity) of the study results  Discussion: paragraph 8 |

*Give information separately for cases and controls in case-control studies and, if applicable, for exposed and unexposed groups in cohort and cross-sectional studies.

**Note:** An Explanation and Elaboration article discusses each checklist item and gives methodological background and published examples of transparent reporting. The STROBE checklist is best used in conjunction with this article (freely available on the Web sites of PLoS Medicine at http://www.plosmedicine.org/, Annals of Internal Medicine at http://www.annals.org/, and Epidemiology at http://www.epidem.com/). Information on the STROBE Initiative is available at www.strobe-statement.org.
