# Supplementary material for: Hospitalizations among adults with chronic kidney disease in the United States: A cohort study
Source: PLoS Med. 2020 Dec 11;17(12):e1003470. doi: 10.1371/journal.pmed.1003470 (PMC7732055; doi:10.1371/journal.pmed.1003470)
Supplement: S2 Table — (DOCX) [file pmed.1003470.s005.docx]

| **S2 Table: Unadjusted rate of all cause, cardiovascular, and non-cardiovascular hospitalizations by key baseline characteristics of CRIC participants (N=3,939).** | | | |
| --- | --- | --- | --- |
|  | **Hospitalization Rate per 100 person-years of follow up (95% CI)** | | |
| **Characteristic** | **All Hospitalizations** | **Cardiovascular Hospitalizations** | **Non-Cardiovascular Hospitalizations** |
| **Overall** | 35.0 (34.3-35.6) | 11.1 (10.8-11.5) | 23.9 (23.3-24.4) |
| **Age, years** |  |  |  |
| 21-44 | 21.9 (20.6-23.2) | 5.2 (4.6-5.9) | 16.6 (15.5-17.8) |
| 45-64 | 34.1 (33.3-35.0) | 10.4 (10.0-10.9) | 23.7 (23.0-24.4) |
| ≥65 | 43.6 (42.3-45.0) | 15.7 (14.9-16.5) | 28.0 (26.9-29.1) |
| **Sex** |  |  |  |
| Male | 33.5 (32.6-34.3) | 11.5 (11.0-12.0) | 22.0 (21.3-22.7) |
| Female | 36.7 (35.7-37.6) | 10.7 (10.2-11.2) | 26.0 (25.2-26.8) |
| **Race/Ethnicity** |  |  |  |
| Non Hispanic White | 30.7 (29.8-31.6) | 8.5 (8.1-9.0) | 22.2 (21.4-22.9) |
| Non Hispanic Black | 41.0 (39.9-42.1) | 14.1 (13.5-14.8) | 26.9 (26.0-27.8) |
| Hispanic | 35.3 (33.3-37.4) | 12.1 (11.0-13.4) | 23.1 (21.5-24.9) |
| Other | 25.2 (22.6-28.0) | 9.1 (7.6-10.9) | 16.1 (14.1-18.4) |
| **Education** |  |  |  |
| Less than HS | 47.7 (45.9-49.6) | 17.2 (16.1-18.3) | 30.6 (29.1-32.0) |
| HS graduation | 40.5 (38.9-42.1) | 13.4 (12.5-14.3) | 27.1 (25.8-28.4) |
| Some College | 39.6 (38.4-40.9) | 12.2 (11.5-12.9) | 27.4 (26.4-28.5) |
| College graduate | 22.5 (21.6-23.3) | 6.3 (5.8-6.7) | 16.2 (15.5-16.9) |
| **Diabetes status** |  |  |  |
| With Diabetes | 47.3 (46.1-48.4) | 15.4 (14.7-16.0) | 31.9 (31.0-32.9) |
| Without Diabetes | 26.3 (25.5-27.0) | 8.1 (7.7-8.5) | 18.2 (17.6-18.8) |
| **Systolic blood pressure** (mmHg) |  |  |  |
| <120 | 30.1 (29.2-31.0) | 9.0 (8.5-9.5) | 21.1 (20.4-21.9) |
| 120 to <130 | 32.4 (31.1-33.8) | 9.6 (8.9-10.3) | 22.8 (21.7-24.0) |
| 130 to <140 | 37.2 (35.6-38.9) | 12.6 (11.7-13.7) | 24.5 (23.2-26.0) |
| ≥140 | 46.0 (44.5-47.7) | 16.0 (15.0-16.9) | 30.1 (28.8-31.4) |
| **eGFR**, ml/min/1.73m^2^ |  |  |  |
| <30 | 51.2 (49.0-53.4) | 17.2 (15.9-18.5) | 34.0 (32.2-35.8) |
| 30 to <45 | 43.5 (42.3-44.8) | 13.9 (13.2-14.7) | 29.6 (28.6-30.7) |
| 45 to <60 | 31.4 (30.3-32.4) | 10.1 (9.5-10.7) | 21.3 (20.4-22.1) |
| ≥60 | 20.7 (19.7-21.8) | 5.8 (5.3-6.3) | 15.0 (14.1-15.8) |
| **Urine protein-creatinine ratio** (mg/g) |  |  |  |
| <150 | 28.6 (27.8-29.3) | 8.9 (8.5-9.3) | 19.7 (19.1-20.3) |
| 150 to <500 | 40.7 (39.1-42.3) | 12.0 (11.2-12.9) | 28.6 (27.3-30.0) |
| ≥500 | 47.6 (45.9-49.3) | 16.3 (15.3-17.3) | 31.3 (29.9-32.7) |
| eGFR – estimated glomerular filtration rate | | | |
