# Supplementary material for: Hospitalizations among adults with chronic kidney disease in the United States: A cohort study
Source: PLoS Med. 2020 Dec 11;17(12):e1003470. doi: 10.1371/journal.pmed.1003470 (PMC7732055; doi:10.1371/journal.pmed.1003470)
Supplement: S3 Table — (DOCX) [file pmed.1003470.s006.docx]

| **S3 Table: Multivariable adjusted rate of all cause, cardiovascular, and non-cardiovascular hospitalizations by age, race/ethnicity, and diabetes of CRIC participants (N=3,939). Rates reported as per 100 person-years.** | | | | | | |
| --- | --- | --- | --- | --- | --- | --- |
|  | **All-Cause**  **Hospitalization** | | **Cardiovascular Hospitalization** | | **Non-Cardiovascular Hospitalization** | |
|  | **Rate (95% CI)** | **p-value** | **Rate (95% CI)** | **p-value** | **Rate (95% CI)** | **p-value** |
| **Age, years** |  | <0.001 |  | <0.001 |  | <0.001 |
| 21-44 | 22.7 (21.2-24.2) |  | 5.7 (5.0-6.5) |  | 16.8 (15.6-18.2) |  |
| 45-64 | 31.4 (30.3-32.5) |  | 10.0 (9.4-10.6) |  | 21.3 (20.3-22.2) |  |
| ≥65 | 39.1 (37.5-40.7) |  | 14.7 (13.7-15.7) |  | 24.4 (23.2-25.7) |  |
| **Race/Ethnicity** |  | <0.001 |  | <0.001 |  | <0.001 |
| Non Hispanic White | 30.1 (29.2-31.1) |  | 7.8 (7.3-8.4) |  | 22.2 (21.4-23.1) |  |
| Non Hispanic Black | 37.7 (36.5-38.9) |  | 12.1 (11.5-12.9) |  | 25.3 (24.3-26.2) |  |
| Hispanic | 30.9 (29.1-32.8) |  | 10.1 (9.1-11.2) |  | 20.6 (19.2-22.2) |  |
| Other | 24.0 (21.5-26.7) |  | 8.2 (6.9-9.9) |  | 15.5 (13.6-17.8) |  |
| **Diabetes status** |  | <0.001 |  | <0.001 |  | <0.001 |
| With Diabetes | 39.4 (37.9-41.0) |  | 12.2 (11.4-13.1) |  | 26.8 (25.6-28.2) |  |
| Without Diabetes | 23.3 (22.3-24.2) |  | 7.3 (6.8-7.8) |  | 15.8 (15.0-16.6) |  |
| Models adjusted for age, race, and diabetes.  CI – confidence interval | | | | | | |
