# Supplementary material for: Hospitalizations among adults with chronic kidney disease in the United States: A cohort study
Source: PLoS Med. 2020 Dec 11;17(12):e1003470. doi: 10.1371/journal.pmed.1003470 (PMC7732055; doi:10.1371/journal.pmed.1003470)
Supplement: S4 Table — (DOCX) [file pmed.1003470.s007.docx]

| **S4 Table: Multivariable adjusted rate ratios of all cause, cardiovascular, and non-cardiovascular hospitalizations by key baseline characteristics of CRIC participants (N=3,939)** | | | | | | |
| --- | --- | --- | --- | --- | --- | --- |
|  | **All-Cause Hospitalizations** | | **Cardiovascular Hospitalizations** | | **Non-Cardiovascular Hospitalizations** | |
|  | **RR (95% CI)** | **p-value** | **RR (95% CI)** | **p-value** | **RR (95% CI)** | **p-value** |
| **Age**, years |  | <0.001 |  | <0.001 |  | <0.001 |
| 21-44 | 0.73 (0.68-0.79) |  | 0.59 (0.51-0.68) |  | 0.79 (0.73-0.86) |  |
| 45-64 | REF |  | REF |  | REF |  |
| ≥65 | 1.21 (1.16-1.26) |  | 1.46 (1.36-1.58) |  | 1.10 (1.05-1.16) |  |
| **Sex** |  | <0.001 |  | 0.002 |  | <0.001 |
| Male | REF |  | REF |  | REF |  |
| Female | 1.08 (1.04-1.13) |  | 0.89 (0.83-0.96) |  | 1.18 (1.13-1.24) |  |
| **Race/Ethnicity** |  | <0.001 |  | <0.001 |  | <0.001 |
| Non Hispanic White | REF |  | REF |  | REF |  |
| Non Hispanic Black | 1.04 (1.00-1.09) |  | 1.25 (1.16-1.35) |  | 0.96 (0.91-1.01) |  |
| Hispanic | 0.86 (0.79-0.94) |  | 1.07 (0.92-1.25) |  | 0.78 (0.70-0.86) |  |
| Other | 0.88 (0.78-0.99) |  | 1.20 (0.99-1.46) |  | 0.76 (0.66-0.88) |  |
| **Diabetes status** |  | <0.001 |  | <0.001 |  | <0.001 |
| With Diabetes | 1.47 (1.42-1.53) |  | 1.43 (1.34-1.54) |  | 1.49 (1.42-1.57) |  |
| Without Diabetes | REF |  | REF |  | REF |  |
| **Systolic blood pressure** (mmHg) |  | <0.001 |  | <0.001 |  | 0.02 |
| <120 | 1.01 (0.96-1.07) |  | 1.04 (0.95-1.15) |  | 1.00 (0.94-1.06) |  |
| 120 to <130 | REF |  | REF |  | REF |  |
| 130 to <140 | 1.05 (0.99-1.12) |  | 1.16 (1.04-1.30) |  | 1.00 (0.93-1.08) |  |
| ≥140 | 1.14 (1.08-1.21) |  | 1.24 (1.12-1.37) |  | 1.09 (1.02-1.17) |  |
| **eGFR**, ml/min/1.73m^2^ |  | <0.001 |  | <0.001 |  | <0.001 |
| <30 | 1.62 (1.50-1.75) |  | 1.76 (1.53-2.02) |  | 1.56 (1.43-1.71) |  |
| 30 to <45 | 1.45 (1.36-1.55) |  | 1.51 (1.34-1.70) |  | 1.43 (1.33-1.55) |  |
| 45 to <60 | 1.16 (1.09-1.23) |  | 1.19 (1.06-1.34) |  | 1.14 (1.06-1.23) |  |
| ≥60 | REF |  | REF |  | REF |  |
| **Urine protein-creatinine ratio** (mg/g) |  | <0.001 |  | <0.001 |  | <0.001 |
| <150 | REF |  | REF |  | REF |  |
| 150 to <500 | 1.26 (1.20-1.32) |  | 1.16 (1.06-1.27) |  | 1.31 (1.23-1.39) |  |
| ≥500 | 1.38 (1.31-1.45) |  | 1.46 (1.34-1.60) |  | 1.34 (1.26-1.42) |  |
| Models adjusted for age, race, sex, clinical center, education, systolic blood pressure, diabetes, urine protein-to-creatinine ratio, CRIC eGFR.  eGFR - estimated glomerular filtration rate; RR -rate ratio; CI – confidence interval | | | | | | |
