# Supplementary material for: Hospitalizations among adults with chronic kidney disease in the United States: A cohort study
Source: PLoS Med. 2020 Dec 11;17(12):e1003470. doi: 10.1371/journal.pmed.1003470 (PMC7732055; doi:10.1371/journal.pmed.1003470)
Supplement: S5 Table — (DOCX) [file pmed.1003470.s008.docx]

| **S5 Table: Multivariable-adjusted all-cause, cardiovascular, and non-cardiovascular hospitalization rates in CRIC participants by proteinuria and eGFR level during the follow-up period.** | | | |
| --- | --- | --- | --- |
|  | **Hospitalization Rate (per 100 person-years)** | | |
|  | **All-Cause** | **Cardiovascular** | **Non-Cardiovascular** |
| **UPCR <150**, mg/g |  |  |  |
| eGFR <30 | 43.0 (39.5-46.8) | 14.1 (12.2-16.4) | 28.3 (25.5-31.3) |
| eGFR 30 to <45 | 31.3 (29.4-33.2) | 9.7 (8.7-10.8) | 21.1 (19.7-22.7) |
| eGFR 45 to <60 | 25.0 (23.6-26.5) | 8.0 (7.2-8.9) | 16.7 (15.5-17.9) |
| eGFR ≥60 | 20.1 (18.8-21.6) | 6.0 (5.3-6.9) | 13.8 (12.7-15.0) |
| **UPCR 150 to <500**, mg/g |  |  |  |
| eGFR <30 | 38.6 (34.7-42.9) | 13.0 (10.8-15.7) | 25.2 (22.1-28.7) |
| eGFR 30 to <45 | 43.3 (40.4-46.4) | 13.3 (11.8-15.1) | 29.3 (27.0-31.9) |
| eGFR 45 to <60 | 29.4 (27.2-31.8) | 7.5 (6.5-8.7) | 21.6 (19.7-23.7) |
| eGFR ≥60 | 31.7 (28.1-35.7) | 8.6 (6.8-10.9) | 22.5 (19.6-25.9) |
| **UPCR ≥500**, mg/g |  |  |  |
| eGFR <30 | 44.9 (41.5-48.7) | 15.2 (13.2-17.4) | 29.2 (26.4-32.2) |
| eGFR 30 to <45 | 41.9 (39.2-44.7) | 13.8 (12.3-15.5) | 27.4 (25.3-29.7) |
| eGFR 45 to <60 | 39.4 (36.3-42.8) | 14.0 (12.1-16.1) | 25.1 (22.6-27.8) |
| eGFR ≥60 | 38.7 (33.7-44.5) | 13.7 (10.8-17.4) | 24.6 (20.7-29.2) |
| Models adjusted for age, race, sex, clinical center, education, systolic blood pressure, diabetes, CRIC eGFR, UPCR and interaction between eGFR*UPCR.  eGFR – estimated glomerular filtration rate (ml/min/1.73m^2^); UPCR- urine protein-creatinine ratio | | | |
