# Supplementary material for: Hospitalizations among adults with chronic kidney disease in the United States: A cohort study
Source: PLoS Med. 2020 Dec 11;17(12):e1003470. doi: 10.1371/journal.pmed.1003470 (PMC7732055; doi:10.1371/journal.pmed.1003470)
Supplement: S6 Table — (DOCX) [file pmed.1003470.s009.docx]

| **S6 Table: Unadjusted rate of all cause, cardiovascular, and non-cardiovascular ≤1 day hospitalizations by key baseline characteristics of CRIC participants (N=3,939).** | | | |
| --- | --- | --- | --- |
|  | **Hospitalization Rate per 100 person-years of follow up (95% CI)** | | |
| **Characteristic** | **All Hospitalizations** | **Cardiovascular Hospitalizations** | **Non-Cardiovascular Hospitalizations** |
| **Overall** | 24.8 (24.3-25.4) | 3.2 (3.0-3.4) | 21.6 (21.1-22.1) |
| **Age, years** |  |  |  |
| 21-44 | 24.5 (23.1-25.9) | 2.6 (2.2-3.1) | 21.9 (20.6-23.3) |
| 45-64 | 25.8 (25.1-26.5) | 3.3 (3.0-3.5) | 22.5 (21.8-23.2) |
| ≥65 | 22.9 (22.0-24.0) | 3.4 (3.0-3.8) | 19.5 (18.7-20.5) |
| **Sex** |  |  |  |
| Male | 23.5 (22.8-24.3) | 3.2 (2.9-3.4) | 20.4 (19.7-21.1) |
| Female | 26.2 (25.4-27.0) | 3.3 (3.0-3.5) | 23.0 (22.2-23.7) |
| **Race/Ethnicity** |  |  |  |
| Non Hispanic White | 20.0 (19.3-20.7) | 2.3 (2.1-2.6) | 17.6 (17.0-18.3) |
| Non Hispanic Black | 32.2 (31.2-33.2) | 4.4 (4.0-4.8) | 27.8 (26.9-28.7) |
| Hispanic | 20.2 (18.7-21.8) | 2.6 (2.1-3.3) | 17.5 (16.1-19.0) |
| Other | 20.1 (17.8-22.6) | 2.8 (2.0-3.8) | 17.3 (15.2-19.7) |
| **Education** |  |  |  |
| Less than HS | 30.9 (29.4-32.3) | 4.4 (3.9-5.0) | 26.5 (25.2-27.9) |
| HS graduation | 27.8 (26.5-29.2) | 3.1 (2.7-3.6) | 24.7 (23.5-26.0) |
| Some College | 29.3 (28.3-30.4) | 3.7 (3.4-4.1) | 25.6 (24.6-26.6) |
| College graduate | 16.8 (16.0-17.5) | 2.2 (2.0-2.5) | 14.5 (13.9-15.2) |
| **Diabetes status** |  |  |  |
| With Diabetes | 28.3 (27.4-29.2) | 3.4 (3.1-3.7) | 24.9 (24.1-25.8) |
| Without Diabetes | 22.3 (21.7-23.0) | 3.1 (2.9-3.3) | 19.2 (18.6-19.9) |
| **Systolic blood pressure** (mmHg) |  |  |  |
| <120 | 22.5 (21.7-23.3) | 2.8 (2.6-3.1) | 19.6 (18.9-20.4) |
| 120 to <130 | 24.0 (22.8-25.1) | 2.6 (2.2-3.0) | 21.4 (20.3-22.5) |
| 130 to <140 | 26.7 (25.4-28.2) | 3.6 (3.1-4.1) | 23.2 (21.9-24.5) |
| ≥140 | 29.0 (27.8-30.3) | 4.3 (3.8-4.8) | 24.8 (23.6-26.0) |
| **eGFR**, ml/min/1.73m^2^ |  |  |  |
| <30 | 26.0 (24.4-27.7) | 4.4 (3.8-5.1) | 21.6 (20.2-23.1) |
| 30 to <45 | 26.5 (25.5-27.5) | 3.1 (2.8-3.5) | 23.4 (22.4-24.3) |
| 45 to <60 | 24.0 (23.1-24.9) | 3.3 (3.0-3.7) | 20.6 (19.8-21.5) |
| ≥60 | 23.2 (22.1-24.3) | 2.5 (2.2-2.9) | 20.7 (19.7-21.7) |
| **Urine protein-creatinine ratio** (mg/g) |  |  |  |
| <150 | 21.9 (21.2-22.5) | 2.8 (2.6-3.0) | 19.1 (18.5-19.7) |
| 150 to <500 | 25.2 (24.0-26.5) | 3.0 (2.6-3.4) | 22.2 (21.1-23.4) |
| ≥500 | 31.0 (29.6-32.4) | 4.6 (4.1-5.1) | 26.4 (25.1-27.7) |
| eGFR – estimated glomerular filtration rate | | | |
