# Supplementary material for: Hospitalizations among adults with chronic kidney disease in the United States: A cohort study
Source: PLoS Med. 2020 Dec 11;17(12):e1003470. doi: 10.1371/journal.pmed.1003470 (PMC7732055; doi:10.1371/journal.pmed.1003470)
Supplement: S7 Table — Rates reported as per 100 person-years. (DOCX) [file pmed.1003470.s010.docx]

| **S7 Table: Multivariable adjusted rates of all cause, cardiovascular, and non-cardiovascular ≤1-day hospitalizations by age, race/ethnicity, and diabetes of CRIC participants (N=3,939). Rates reported as per 100 person-years.** | | | | | | |
| --- | --- | --- | --- | --- | --- | --- |
|  | **All-Cause**  **Hospitalization Rate** | | **Cardiovascular Hospitalization Rate** | | **Non-Cardiovascular Hospitalization Rate** | |
|  | **Rate (95% CI)** | **p-value** | **Rate (95% CI)** | **p-value** | **Rate (95% CI)** | **p-value** |
| **Age, years** |  | <0.001 |  | <0.05 |  | <0.001 |
| 21-44 | 23.8 (22.3-25.4) |  | 2.4 (2.0-3.0) |  | 21.4 (19.9-22.9) |  |
| 45-64 | 23.3 (22.3-24.3) |  | 3.0 (2.7-3.4) |  | 20.3 (19.4-21.2) |  |
| ≥65 | 20.7 (19.6-21.8) |  | 3.2 (2.8-3.6) |  | 17.5 (16.5-18.6) |  |
| **Race/Ethnicity** |  | <0.001 |  | <0.001 |  | <0.001 |
| Non-Hispanic White | 20.5 (19.7-21.3) |  | 2.3 (2.0-2.5) |  | 18.2 (17.5-19.0) |  |
| Non-Hispanic Black | 32.2 (31.1-33.3) |  | 4.2 (3.8-4.6) |  | 27.9 (26.9-28.9) |  |
| Hispanic | 19.5 (18.1-21.1) |  | 2.6 (2.1-3.2) |  | 16.9 (15.6-18.4) |  |
| Other | 20.1 (17.8-22.6) |  | 2.7 (2.0-3.7) |  | 17.3 (15.2-19.7) |  |
| **Diabetes status** |  | <0.001 |  | 0.90 |  | <0.001 |
| With Diabetes | 25.2 (24.0-26.4) |  | 2.9 (2.5-3.3) |  | 22.3 (21.2-23.4) |  |
| Without Diabetes | 20.2 (19.3-21.1) |  | 2.8 (2.5-3.2) |  | 17.3 (16.5-18.2) |  |
| Models adjusted for age, race, and diabetes. | | | | | | |
