# Supplementary material for: Hospitalizations among adults with chronic kidney disease in the United States: A cohort study
Source: PLoS Med. 2020 Dec 11;17(12):e1003470. doi: 10.1371/journal.pmed.1003470 (PMC7732055; doi:10.1371/journal.pmed.1003470)
Supplement: S8 Table — (DOCX) [file pmed.1003470.s011.docx]

| **S8 Table: Multivariable adjusted rate ratios of all cause, cardiovascular, and non-cardiovascular ≤1-day hospitalizations by key baseline characteristics of CRIC participants (N=3,939).** | | | | | | |
| --- | --- | --- | --- | --- | --- | --- |
|  | **All-Cause Hospitalizations** | | **Cardiovascular Hospitalizations** | | **Non-Cardiovascular Hospitalizations** | |
|  | **RR (95% CI)** | **p-value** | **RR (95% CI)** | **p-value** | **RR (95% CI)** | **p-value** |
| **Age, years** |  | 0.06 |  | 0.05 |  | 0.02 |
| 21-44 | 0.95 (0.89-1.02) |  | 0.83 (0.67-1.02) |  | 0.97 (0.90-1.05) |  |
| 45-64 | REF |  | REF |  | REF |  |
| ≥65 | 0.94 (0.89-1.00) |  | 1.11 (0.96-1.29) |  | 0.92 (0.87-0.98) |  |
| **Sex** |  | 0.002 |  | 0.66 |  | <0.001 |
| Male | REF |  | REF |  | REF |  |
| Female | 1.08 (1.03-1.13) |  | 0.97 (0.85-1.11) |  | 1.09 (1.04-1.15) |  |
| **Race/Ethnicity** |  | <0.001 |  | <0.001 |  | <0.001 |
| Non-Hispanic White | REF |  | REF |  | REF |  |
| Non-Hispanic Black | 1.40 (1.33-1.48) |  | 1.64 (1.41-1.90) |  | 1.37 (1.30-1.45) |  |
| Hispanic | 0.95 (0.86-1.06) |  | 1.01 (0.75-1.36) |  | 0.95 (0.84-1.06) |  |
| Other | 0.99 (0.87-1.13) |  | 1.24 (0.88-1.75) |  | 0.96 (0.83-1.10) |  |
| **Diabetes status** |  | <0.001 |  | 0.24 |  | <0.001 |
| With Diabetes | 1.16 (1.11-1.22) |  | 0.92 (0.81-1.05) |  | 1.20 (1.14-1.26) |  |
| Without Diabetes | REF |  | REF |  | REF |  |
| **Systolic blood pressure** (mmHg) |  | 0.03 |  | 0.01 |  | 0.21 |
| <120 | 1.03 (0.97-1.10) |  | 1.21 (1.01-1.45) |  | 1.01 (0.94-1.08) |  |
| 120 to <130 | REF |  | REF |  | REF |  |
| 130 to <140 | 1.08 (1.00-1.16) |  | 1.32 (1.07-1.64) |  | 1.05 (0.96-1.13) |  |
| ≥140 | 1.11 (1.03-1.19) |  | 1.37 (1.12-1.66) |  | 1.07 (1.00-1.16) |  |
| **eGFR**, ml/min/1.73m^2^ |  | 0.23 |  | 0.001 |  | 0.04 |
| <30 | 1.04 (0.95-1.14) |  | 1.50 (1.18-1.92) |  | 0.98 (0.89-1.08) |  |
| 30 to <45 | 1.07 (1.00-1.15) |  | 1.08 (0.88-1.33) |  | 1.07 (0.99-1.15) |  |
| 45 to <60 | 1.02 (0.95-1.09) |  | 1.24 (1.03-1.49) |  | 0.99 (0.92-1.06) |  |
| ≥60 | REF |  | REF |  | REF |  |
| **Urine protein-creatinine ratio** (mg/g) |  | <0.001 |  | <0.001 |  | <0.001 |
| <150 | REF |  | REF |  | REF |  |
| 150 to <500 | 1.09 (1.02-1.15) |  | 1.01 (0.85-1.19) |  | 1.10 (1.03-1.17) |  |
| ≥500 | 1.31 (1.23-1.39) |  | 1.53 (1.29-1.80) |  | 1.28 (1.20-1.37) |  |
| Models adjusted for age, race, sex, clinical center, education, systolic blood pressure, diabetes, urine protein-creatinine ratio, CRIC eGFR  eGFR - estimated glomerular filtration rate; RR - rate ratio; CI - confidence interval. | | | | | | |
