# Supplementary material for: Hospitalizations among adults with chronic kidney disease in the United States: A cohort study
Source: PLoS Med. 2020 Dec 11;17(12):e1003470. doi: 10.1371/journal.pmed.1003470 (PMC7732055; doi:10.1371/journal.pmed.1003470)
Supplement: S9 Table — (DOCX) [file pmed.1003470.s012.docx]

| **S9 Table: Multivariable-adjusted all-cause, cardiovascular, and non-cardiovascular ≤1 day hospitalization rates in CRIC participants by proteinuria and eGFR level during the follow-up period.** | | | |
| --- | --- | --- | --- |
|  | **Hospitalization Rate (per 100 person-years)** | | |
|  | **All-Cause** | **Cardiovascular^** | **Non-Cardiovascular** |
| **UPCR <150**, mg/g |  |  |  |
| eGFR <30 | 23.3 (20.7-26.2) | 3.5 (2.6-4.8) | 19.7 (17.3-22.3) |
| eGFR 30 to <45 | 22.1 (20.6-23.8) | 2.2 (1.7-2.7) | 19.9 (18.5-21.5) |
| eGFR 45 to <60 | 20.6 (19.2-22.0) | 3.0 (2.5-3.5) | 17.5 (16.3-18.8) |
| eGFR ≥60 | 19.6 (18.2-21.0) | 2.1 (1.7-2.6) | 17.4 (16.1-18.8) |
| **UPCR 150 to <500**, mg/g |  |  |  |
| eGFR <30 | 24.6 (21.5-28.2) | 3.7 (2.6-5.3) | 20.8 (17.9-24.1) |
| eGFR 30 to <45 | 23.2 (21.1-25.4) | 2.6 (2.0-3.4) | 20.6 (18.6-22.7) |
| eGFR 45 to <60 | 21.4 (19.5-23.4) | 2.4 (1.8-3.1) | 18.9 (17.2-20.9) |
| eGFR ≥60 | 24.3 (21.3-27.6) | 2.4 (1.6-3.6) | 21.7 (18.9-24.9) |
| **UPCR ≥500**, mg/g |  |  |  |
| eGFR <30 | 24.8 (22.3-27.6) | 4.7 (3.6-6.0) | 20.0 (17.8-22.6) |
| eGFR 30 to <45 | 28.0 (25.9-30.3) | 4.0 (3.2-4.9) | 24.0 (22.0-26.1) |
| eGFR 45 to <60 | 29.1 (26.5.-32.0) | 3.8 (2.9-4.9) | 25.3 (22.8-28.0) |
| eGFR ≥60 | 30.2 (26.0-35.1) | 4.0 (2.7-6.1) | 26.0 (22.2-30.6) |
| Models adjusted for age, race, sex, clinical center, education, systolic blood pressure, diabetes, CRIC eGFR, UPCR and interaction between eGFR*UPCR.  eGFR – estimated glomerular filtration rate (ml/min/1.73m^2^); UPCR- urine protein-creatinine ratio  ^interaction term not statistically significant in the cardiovascular hospitalization model. | | | |
