# Supplementary material for: Hospitalizations among adults with chronic kidney disease in the United States: A cohort study
Source: PLoS Med. 2020 Dec 11;17(12):e1003470. doi: 10.1371/journal.pmed.1003470 (PMC7732055; doi:10.1371/journal.pmed.1003470)
Supplement: S1 Text — (DOCX) [file pmed.1003470.s013.docx]

**S1 Text: Categories of CCS**

| Multi-level CCS Diagnosis Category | Single-Level CCS Diagnosis Category |
| --- | --- |
| Infectious and parasitic infections | Tuberculosis [1] Septicemia (except in labor) [2] Streptococcal septicemia Staphylococcal septicemia E Coli septicemia Other gram negative septicemia Other specified septicemia Unspecified septicemia Sexually transmitted infections (not HIV or hepatitis) [9] Other bacterial infections [3] Mycoses [4] Candidiasis of the mouth (thrush) Other mycoses Viral infection HIV infection [5] Hepatitis [6] Other viral infections [7] Herpes zoster infection Herpes simplex infection Other and unspecified viral infection Other infections; including parasitic [8] Immunizations and screening for infectious disease [10] |
| Neoplasms | Cancer of colon [14] Cancer of rectum and anus [15] Other gastrointestinal cancer Cancer of esophagus [12] Cancer of stomach [13] Cancer of liver and intrahepatic bile duct [16] Cancer of pancreas [17] Cancer of other GI organs; peritoneum [18] Cancer of bronchus; lung [19] Cancer of skin; Melanomas of skin [22] Other non-epithelial cancer of skin [23] Cancer of breast [24] Cancer of uterus and cervix Cancer of uterus [25] Cancer of cervix [26] Cancer of ovary and other female genital organs Cancer of ovary [27] Cancer of other female genital organs [28] Cancer of male genital organs Cancer of prostate [29] Cancer of testis [30] Cancer of other male genital organs [31] Cancer of urinary organs Cancer of bladder [32] Cancer of kidney and renal pelvis [33] Cancer of other urinary organs [34] Cancer of lymphatic and hematopoietic tissue Hodgkins disease [37] Non-Hodgkins lymphoma [38] Leukemias [39] Multiple myeloma [40] Cancer; other primary Cancer of head and neck [11] Cancer; other respiratory and intrathoracic [20] Cancer of bone and connective tissue [21] Cancer of brain and nervous system [35] Cancer of thyroid [36] Cancer; other and unspecified primary [41] Secondary malignancies [42] Secondary malignancy of lymph nodes; Secondary malignancy of lung Secondary malignancy of liver Secondary malignancy of brain/spine Secondary malignancy of bone Other secondary malignancy Malignant neoplasm without specification of site [43] Neoplasms of unspecified nature or uncertain behavior [44] Maintenance chemotherapy; radiotherapy [45] Radiotherapy Chemotherapy Benign neoplasms Benign neoplasm of uterus [46] Other and unspecified benign neoplasm [47] Benign neoplasm of ovary Benign neoplasm of colon Benign neoplasm of the thyroid Benign neoplasm of cerebral meninges Other and unspecified benign neoplasms |
| Endocrine, nutrition, metabolic-related causes | Thyroid disorders [48] Thyrotoxicosis with or without goiter Other thyroid disorders Diabetes mellitus without complication [49] Diabetes mellitus with complications [50] Diabetes with ketoacidosis or uncontrolled diabetes Diabetes with renal manifestations Diabetes with ophthalmic manifestations Diabetes with neurological manifestations Diabetes with circulatory manifestations Diabetes with unspecified complications Diabetes with other manifestations Other endocrine disorders [51] Nutritional deficiencies [52] Unspecified protein-calorie malnutrition Other malnutrition Disorders of lipid metabolism [53] Gout and other crystal arthropathies [54] Fluid and electrolyte disorders [55] Hyposmolality Hypovolemia Hyperpotassemia Hypopotassemia Other fluid and electrolyte disorders Cystic fibrosis [56] Immunity disorders [57] Other nutritional; endocrine; and metabolic disorders [58] Disorders of mineral metabolism Obesity Other and unspecified metabolic; nutritional; and endocrine disorders |
| Diseases of the blood and blood-forming organs | Anemia Acute posthemorrhagic anemia [60] Sickle cell anemia [61] Deficiency and other anemia [59] Iron deficiency Anemia Other deficiency anemia Aplastic anemia Chronic blood loss anemia Acquired hemolytic anemia Other specified anemia; unspecified Coagulation and hemorrhagic disorders [62] Coagulation defects Thrombocytopenia Other coagulation and hemorrhagic disorders Diseases of white blood cells [63] Other hematologic conditions [64] |
| Mental Illness | Adjustment disorders [650] Anxiety disorders [651] Attention deficit, conduct, and disruptive behavior disorders [652] Conduct disorder [6521] Oppositional defiant disorder [6522] Attention deficit disorder and Attention deficit hyperactivity disorder [6523] Delirium, dementia, and amnestic and other cognitive disorders [653] Developmental disorders [654] Communication disorders [6541] Developmental disabilities [6542] Intellectual disabilities [6543] Learning disorders [6544] Motor skill disorders [6545] Disorders usually diagnosed in infancy, childhood, or adolescence [655] Elimination disorders [6551] Other disorders of infancy childhood or adolescence [6552] Pervasive developmental disorders [6553] Tic disorders [6554] Impulse control disorders not elsewhere classified [656] Mood disorders [657] Bipolar disorders [6571] Depressive disorders [6572] Personality disorders [658] Schizophrenia and other psychotic disorders [659] Alcohol-related disorders [660] Substance-related disorders [661] Suicide and intentional self-inflicted injury [662] Screening and history of mental health and substance abuse codes [663] Codes related to mental health disorders [6631] Codes related to substance-related disorders [6632] Miscellaneous mental health disorders [670] Dissociative disorders [6701] Eating disorders [6702] Factitious disorders [6703] Psychogenic disorders [6704] Sexual and gender identity disorders [6705] Sleep disorders [6706] Somatoform disorders [6707] Mental disorders due to general medical conditions not elsewhere classified [6708] Other miscellaneous mental conditions [6709] |
| Diseases of the nervous system and sense organs | Central nervous system infection Meningitis (except that caused by TB or STD) [76] Encephalitis (except that caused by TB or STD) [77] Other CNS infection and poliomyelitis [78] Hereditary and degenerative nervous system conditions Parkinsons disease [79] Multiple sclerosis [80] Other hereditary and degenerative nervous system conditions [81] Disorders of the autonomic nervous system Other and unspecified hereditary and degenerative nervous conditions Paralysis [82] Hemiplegia Other paralysis Epilepsy; convulsions [83] Epilepsy Convulsions Headache; including migraine [84] Migraine Other headache Coma; stupor; and brain damage [85] Eye disorders Cataract [86] Retinal detachments; defects; vascular occlusion; and retinopathy [87] Retinal detachment with defect Other retinal detachment or defect Other retinal disorders Glaucoma [88] Blindness and vision defects [89] Inflammation; infection of eye (except that caused by TB or STD) [90] Other eye disorders [91] Ear conditions Otitis media and related conditions [92] Suppurative and unspecified otitis media; Other otitis media and related conditions; Conditions associated with dizziness or vertigo [93] Other ear and sense organ disorders [94] Other nervous system disorders [95] Disorders of the peripheral nervous system Other central nervous system disorders Other nervous system symptoms and disorders |
| Diseases of the circulatory system | Hypertension Essential hypertension [98] Hypertension with complications and secondary hypertension [99] Hypertensive heart and/or renal disease Other hypertensive complications Diseases of the heart Heart valve disorders [96] Chronic rheumatic disease of the heart valves Nonrheumatic mitral valve disorders Nonrheumatic aortic valve disorders Other heart valve disorders Peri-; endo-; and myocarditis; cardiomyopathy (except that caused by TB or STD) [97] Cardiomyopathy Other peri-; endo-; and myocarditis Acute myocardial infarction [100] Coronary atherosclerosis and other heart disease [101] Angina pectoris Unstable angina (intermediate coronary syndrome) Other acute and subacute forms of ischemic heart disease Coronary atherosclerosis Other forms of chronic heart disease Nonspecific chest pain [102] Pulmonary heart disease [103] Other and ill-defined heart disease [104] Conduction disorders [105] Atrioventricular block Bundle branch block Anomalous atrioventricular excitation Other conduction disorders Cardiac dysrhythmias [106] Paroxysmal supraventricular tachycardia Paroxysmal ventricular tachycardia Atrial fibrillation Atrial flutter Premature beats Sinoatrial node dysfunction Other cardiac dysrhythmias Cardiac arrest and ventricular fibrillation [107] Congestive heart failure; nonhypertensive [108] Congestive heart failure Heart failure Cerebrovascular disease Acute cerebrovascular disease [109] Intracranial hemorrhage Occlusion of cerebral arteries Acute but ill-defined cerebrovascular accident Occlusion or stenosis of precerebral arteries [110] Other and ill-defined cerebrovascular disease [111] Transient cerebral ischemia [112] Late effects of cerebrovascular disease [113] Diseases of arteries; arterioles; and capillaries; Peripheral and visceral atherosclerosis [114] Atherosclerosis of arteries of extremities Peripheral vascular disease unspecified Other peripheral and visceral atherosclerosis Aortic; peripheral; and visceral artery aneurysms [115] Abdominal aortic aneurysm; without rupture Other aneurysm Aortic and peripheral arterial embolism or thrombosis [116] Arterial embolism and thrombosis of lower extremity artery Other arterial embolism and thrombosis; Other circulatory disease [117] Hypotension Other and unspecified circulatory disease Diseases of veins and lymphatics Phlebitis; thrombophlebitis and thromboembolism [118] Phlebitis and thrombophlebitis Other venous embolism and thrombosis Varicose veins of lower extremity [119] Hemorrhoids [120] Other diseases of veins and lymphatics [121] |
| Diseases of the Respiratory Tract | Respiratory infections Pneumonia (except that caused by TB or STD) [122] Pneumococcal pneumonia Other bacterial pneumonia Pneumonia; organism unspecified Other pneumonia Influenza [123] Acute and chronic tonsillitis [124] Acute bronchitis [125] Other upper respiratory infections [126] Acute upper respiratory infections of multiple or unspecified sites Chronic sinusitis Croup Other and unspecified upper respiratory infections Chronic obstructive pulmonary disease and bronchiectasis [127] Emphysema Chronic airway obstruction; not otherwise specified Obstructive chronic bronchitis Other chronic pulmonary disease Asthma [128] Chronic obstructive asthma Chronic obstructive asthma without status asthmaticus or exacerbation Chronic obstructive asthma with status asthmaticus Chronic obstructive asthma with acute exacerbation Other and unspecified asthma Other asthma without status asthmaticus or exacerbation Other asthma with status asthmaticus Other asthma with acute exacerbation Aspiration pneumonitis; food/vomitus [129] Pleurisy; pneumothorax; pulmonary collapse [130] Pleurisy; pleural effusion Pulmonary collapse; interstitial and compensatory emphysema Empyema and pneumothorax Respiratory failure; insufficiency; arrest (adult) [131] Respiratory failure Other respiratory insufficiency Lung disease due to external agents [132] Other lower respiratory disease [133] Postinflammatory pulmonary fibrosis, Painful respiration; Other and unspecified lower respiratory disease Other upper respiratory disease [134] |
| Diseases of Digestive System | Intestinal infection [135] Disorders of teeth and jaw [136] Diseases of mouth; excluding dental [137] Upper gastrointestinal disorders Esophageal disorders [138] Esophagitis Other esophageal disorders Gastroduodenal ulcer (except hemorrhage) [139] Gastric ulcer Duodenal ulcer Peptic ulcer; site unspecified Gastrojejunal ulcer Gastritis and duodenitis [140] Acute gastritis Other specified gastritis Unspecified gastritis and gastroduodenitis Duodenitis Other disorders of stomach and duodenum [141] Abdominal hernia [143] Inguinal hernia Inguinal hernia with obstruction or gangrene Inguinal hernia without obstruction or gangrene Diaphragmatic hernia Other abdominal hernia Femoral hernia with obstruction/gangrene Femoral hernia without obstruction/gangrene Umbilical hernia with obstruction/gangrene Umbilical hernia without obstruction/gangrene Ventral hernia with obstruction/gangrene Ventral hernia without obstruction/gangrene Incisional hernia with obstruction/gangrene Incisional hernia without obstruction/gangrene Other and unspecified hernia Lower gastrointestinal disorders Appendicitis and other appendiceal conditions [142] Acute appendicitis with abscess or peritonitis Acute appendicitis without abscess or peritonitis Acute appendicitis; not otherwise specified Other appendiceal conditions Regional enteritis and ulcerative colitis [144] Intestinal obstruction without hernia [145] Paralytic ileus Impaction of intestine Peritoneal or intestinal adhesions Other intestinal obstruction Diverticulosis and diverticulitis [146] Diverticulosis Diverticulitis Anal and rectal conditions [147] Peritonitis and intestinal abscess [148] Biliary tract disease [149] Cholelithiasis with acute cholecystitis, Cholelithiasis with other cholecystitis ,Cholelithiasis without mention of cholecystitis, Calculus of bile duct Cholecystitis without cholelithiasis, Other biliary tract disease Liver disease ;Liver disease; alcohol-related [150] Other liver diseases [151] Cirrhosis of liver without mention of alcohol Liver abscess and sequelae of chronic liver disease Ascites Other and unspecified liver disorders Pancreatic disorders (not diabetes) [152] Acute pancreatitis Chronic pancreatitis Other pancreatic disorders Gastrointestinal hemorrhage [153] Hemorrhage from gastrointestinal ulcer Melena Gastroesophageal laceration syndrome Other esophageal bleeding Hemorrhage of rectum and anus Hematemesis Hemorrhage of gastrointestinal tract Noninfectious gastroenteritis [154] Other gastrointestinal disorders [155] Constipation Dysphagia Other and unspecified gastrointestinal disorders |
| Diseases of the Genitourinary System | Diseases of the urinary system: Nephritis; nephrosis; renal sclerosis [156] Acute and unspecified renal failure [157] Acute renal failure Unspecified renal failure Chronic renal failure [158] Urinary tract infections [159] Infections of kidney Cystitis and urethritis Urinary tract infection; site not specified Calculus of urinary tract [160] Calculus of kidney Calculus of ureter Other and unspecified urinary calculus Other diseases of kidney and ureters [161] Hydronephrosis Other and unspecified diseases of kidney and ureters Other diseases of bladder and urethra [162] Bladder neck obstruction Other and unspecified diseases of bladder and urethra Genitourinary symptoms and ill-defined conditions [163] Hematuria Retention of urine Other and unspecified genitourinary symptoms;  Diseases of male genital organs: Hyperplasia of prostate [164] Inflammatory conditions of male genital organs [165] Other male genital disorders [166]  Diseases of female genital organs: Nonmalignant breast conditions [167] Inflammatory diseases of female pelvic organs [168] Pelvic peritoneal adhesions Cervicitis and endocervicitis Pelvic inflammatory disease (PID) Other inflammatory diseases of female pelvic organs Endometriosis [169] Prolapse of female genital organs [170] Menstrual disorders [171] Ovarian cyst [172] Menopausal disorders [173] Female infertility [174] Other female genital disorders [175] Female genital pain and other symptoms Other and unspecified female genital disorders |
| Complications of pregnancy; childbirth; and the puerperium; congenital anomalies; certain conditions originating in the perinatal period | Contraceptive and procreative management [176] Sterilization Other contraceptive and procreation management Abortion-related disorders Spontaneous abortion [177] Induced abortion [178] Postabortion complications [179] Complications mainly related to pregnancy Ectopic pregnancy [180] Hemorrhage during pregnancy; abruptio placenta; placenta previa [182] Placenta previa Abruptio placenta Other hemorrhage during pregnancy; childbirth and the puerperium Hypertension complicating pregnancy; childbirth and the puerperium [183] Preeclampsia and eclampsia Other hypertension in pregnancy Early or threatened labor [184] Threatened premature labor Early onset of delivery Other early or threatened labor Prolonged pregnancy [185] Diabetes or abnormal glucose tolerance complicating pregnancy; childbirth; or the puerperium [186] Other complications of pregnancy [181] Infections of genitourinary tract during pregnancy, Anemia during pregnancy Mental disorders during pregnancy Missed abortion Hyperemesis gravidarum Infectious and parasitic complications in mother affecting pregnancy Other and unspecified complications of pregnancy Indications for care in pregnancy; labor; and delivery Malposition; malpresentation [187] Breech presentation Other malposition; malpresentation Fetopelvic disproportion; obstruction [188] Fetopelvic disproportion Other disproportion or obstruction Previous cesarean section [189] Fetal distress and abnormal forces of labor [190] Fetal distress Uterine inertia, Precipitate labor Other abnormal forces of labor Polyhydramnios and other problems of amniotic cavity [191] Premature rupture of membranes Infection of amniotic cavity Other problems of amniotic cavity Complications during labor Umbilical cord complication [192] Cord around neck with compression Other and unspecified cord entanglement with or without compression Other umbilical cord complications Trauma to perineum and vulva [193] First degree perineal laceration Second degree perineal laceration Third degree perineal laceration Fourth degree perineal laceration Other perineal laceration and trauma Forceps delivery [194] Other complications of birth; puerperium affecting management of mother [195] Postpartum hemorrhage Complications of the puerperium Cervical incompetence Rhesus isoimmunization Intrauterine death; Failed induction Other obstetrical trauma Other and unspecified complications of birth; puerperium affecting management of mother Normal pregnancy and/or delivery [196] Normal delivery Multiple gestation Outcome of delivery (V codes)  Cardiac and circulatory congenital anomalies [213] Transposition of great vessels Tetralogy of Fallot Ventricular septal defect Atrial septal defect Endocardial cushion defects Pulmonary valve atresia and stenosis Aortic valve stenosis Patent ductus arteriosus Coarctation of aorta Pulmonary artery anomalies Cerebrovascular anomalies Other cardiac and circulatory congenital anomalies Digestive congenital anomalies [214] Esophageal atresia/tracheoesophageal fistula Pyloric stenosis Rectal and large intestine atresia/stenosis Hirshsprungs disease Other digestive congenital anomalies Genitourinary congenital anomalies [215] Undescended testicle Hypospadias and epispadias Obstructive genitourinary defect Other genitourinary congenital anomalies Nervous system congenital anomalies [216] Spina bifida Congenital hydrocephalus Other nervous system congenital anomalies Other congenital anomalies [217] Cleft palate without cleft lip Cleft lip with or without cleft palate Congenital hip dislocation All other congenital anomalies  Liveborn [218] Short gestation; low birth weight; and fetal growth retardation [219] Intrauterine hypoxia and birth asphyxia [220] Respiratory distress syndrome [221] Hemolytic jaundice and perinatal jaundice [222] Birth trauma [223] Other perinatal conditions [224] Respiratory conditions of fetus and newborn; other than respiratory distress Infections specific to the perinatal period Endocrine and metabolic disturbances of fetus and newborn Other and unspecified perinatal condition |
| Diseases of skin and subcutaneous tissues | Skin and subcutaneous tissue infections [197] Cellulitis and abscess Cellulitis and abscess of fingers and toes Cellulitis and abscess of face Cellulitis and abscess of arm Cellulitis and abscess of hand Cellulitis and abscess of leg Cellulitis and abscess of foot Other cellulitis and abscess Other skin and subcutaneous infections Other inflammatory condition of skin [198] Chronic ulcer of skin [199] Decubitus ulcer Chronic ulcer of leg or foot Other chronic skin ulcer Other skin disorders [200] |
| Diseases of the musculoskeletal system and connective tissue | Infective arthritis and osteomyelitis (except that caused by TB or STD) [201] Non-traumatic joint disorders Rheumatoid arthritis and related disease [202] Osteoarthritis [203] Osteoarthritis; localized Osteoarthritis; generalized and unspecified Other non-traumatic joint disorders [204] Spondylosis; intervertebral disc disorders; other back problems [205] Spondylosis and allied disorders Intervertebral disc disorders Other back problems Cervical radiculitis Spinal stenosis; lumbar region Lumbago Sciatica Thoracic or lumbosacral neuritis or radiculitis; unspecified Backache; unspecified Other back pain and disorders Osteoporosis [206] Pathological fracture [207] Acquired deformities Acquired foot deformities [208] Other acquired deformities [209] Systemic lupus erythematosus and connective tissue disorders [210] Other connective tissue disease [211] Other bone disease and musculoskeletal deformities [212] |
| Injuries and poisonings | Joint disorders and dislocations; trauma-related [225] Fractures Fracture of neck of femur (hip) [226] Skull and face fractures [228] Fracture of upper limb [229] Fracture of humerus Fracture of radius and ulna Other fracture of upper limb Fracture of lower limb [230] Fracture of tibia and fibula Fracture of ankle Other fracture of lower limb Other fractures [231] Fracture of vertebral column without mention of spinal cord injury Fracture of ribs; closed Fracture of pelvis Other and unspecified fracture Spinal cord injury [227] Intracranial injury [233] Concussion Other intracranial injury Crushing injury or internal injury [234] Open wounds Open wounds of head; neck; and trunk [235] Open wounds of extremities [236] Sprains and strains [232] Superficial injury; contusion [239] Burns [240] Complications Complication of device; implant or graft [237] Malfunction of device; implant; and graft Infection and inflammation--internal prosthetic device; implant; and graft Other complications of internal prosthetic device; implant; and graft Complications of transplants and reattached limbs Complications of surgical procedures or medical care [238] Cardiac complications Respiratory complications Gastrointestinal complications Urinary complications Hemorrhage or hematoma complicating a procedure Postoperative infection Other complications of surgical and medical procedures Poisoning Poisoning by psychotropic agents [241] Poisoning by other medications and drugs [242] Poisoning by nonmedicinal substances [243] Other injuries and conditions due to external causes [244] |
| Symptoms; signs; and ill-defined conditions and factors influencing health status | Syncope [245] Fever of unknown origin [246] Lymphadenitis [247] Gangrene [248] Shock [249] Nausea and vomiting [250] Abdominal pain [251] Malaise and fatigue [252] Allergic reactions [253] Factors influencing health care Rehabilitation care; fitting of prostheses; and adjustment of devices [254] Administrative/social admission [255] Medical examination/evaluation [256] Other aftercare [257] Other screening for suspected conditions (not mental disorders or infectious disease) [258] |
| Residual Codes | Residual codes; unclassified; all E codes [259 and 260] |
