# Supplementary material for: Hospitalizations among adults with chronic kidney disease in the United States: A cohort study
Source: PLoS Med. 2020 Dec 11;17(12):e1003470. doi: 10.1371/journal.pmed.1003470 (PMC7732055; doi:10.1371/journal.pmed.1003470)
Supplement: S2 Text — (DOCX) [file pmed.1003470.s014.docx]

**S2 Text: Analytical Planning Document**

|  | |
| --- | --- |
| Original CRIC Study Manuscript Planning Document  ***Summary Information*** | |
| **Full Proposal Title** | Hospitalization experience of adults with chronic kidney disease: Findings from the Chronic Renal Insufficiency Cohort (CRIC) Study |
| **Abbreviated Title** | Hospitalizations in CRIC |
| **Abstract/Brief Description** | This will primarily be a descriptive analysis describing the burden and types of hospitalization of all participants enrolled in the original CRIC cohort. |
| **Timing for Data Analysis** | Data analyses can begin immediately |
| **Keywords and Domain** | hospitalization; chronic kidney disease; morbidity |
| ***Proposal Details*** | |
| **Introduction**  (Brief rationale and background) | The prevalence of chronic kidney disease (CKD) is high, and it affects up to 13% of Americans, or around 26 million people, older than 20 years old (1,2). CKD accounts for over $44 billion, or 17%, of Medicare spending alone (2). Patients with CKD experience a significant burden of morbidity and mortality, with an estimated 59% higher mortality rate and 38% higher hospitalization rate than the general population (2). These hospitalizations are more likely to lead to further complications, including re-hospitalizations and death, with worsening kidney function associated with worse outcomes (3,4).  The burden and types of hospitalizations in patients with CKD has not been well characterized in the literature, particularly in patients who have not yet developed end stage renal disease (ESRD). While CKD has been shown in individual studies to be a known risk factor for specific diseases such as cardiovascular events (5–9), infections (10), and GI bleeds (11), few studies have looked more generally at all causes of hospitalizations, especially non-cardiovascular causes of hospitalization. Previous studies have also been limited by including patients from only one center (12) or one region (3), studying only the elderly (4), looking primarily at patients with ESRD but not earlier stages of CKD (13), and studying primarily claims data retrospectively (3,4,12,13).  The Chronic Renal Insufficiency Cohort (CRIC) is a 7 center cohort study that has followed patients with CKD to identify risk factors for progression of CKD and cardiovascular disease (CVD) (14,15). This cohort has collected data on patients prospectively and has included patients with earlier stages of CKD. It also may be more generalizable as it includes patients from clinical centers across the country (14). Using CRIC data, we plan to characterize the burden and types hospitalizations by CKD patients, both cardiovascular and non-cardiovascular, and hope to examine the effect of severity of kidney dysfunction, level of proteinuria, and diabetes status on the risks of hospitalization. We hypothesize that in addition to cardiovascular hospitalizations, there is a substantial burden of non-cardiovascular hospitalizations in patients with CKD. We also hypothesize that severity of CKD, proteinuria, age, and diabetes status tracks frequency and severity of hospitalization. |
| **Research Hypothesis**  (Clear statement of scientific questions to be addressed) | 1. There is a large burden of hospitalizations, particularly non-cardiovascular hospitalizations, in patients with CKD 2. The proportion of hospitalizations with adjudicated cardiovascular events increases with increasing severity of CKD, proteinuria, age, and among those with diabetes. 3. Severity of CKD, proteinuria, age and diabetes status are associated independently with increased frequency and severity of hospitalization (as measured by length of hospital stay and in-hospital death) 4. Leading type of hospitalization (as indicated by the primary ICD-9 code) differs by level of eGFR, proteinuria, age and diabetes status.    1. The leading type of hospitalization among participants with diabetes will be endocrine-related, while older participants and those with the worst kidney function and highest levels of proteinuria will have more CVD-related hospitalizations compared to their counterparts. |
| **Data**  (List of variables to be used, biological samples including volume of samples, if relevant) | Primary Predictors/Exposures: eGFR level, proteinuria, age, diabetes |
|  | Covariates: gender, race/ethnicity, socioeconomic parameters, ABI, BMI, history of CVD at baseline, |
|  | Outcomes: Hospitalization rate, hospitalization length of stay (date of discharge minus date of admission), Classification of hospitalization using the Clinical Classification System (see below for a more detailed description), in-hospital death |
|  | Effect Modifiers: |
|  | Other: |
| ***Are these data currently available?*** | Yes  No |
| **Analysis Plans and Methods in consultation with the SDCC**  (Detailed description of proposed statistical analyses. **Please specify whether the analysis is cross-sectional or longitudinal)** | Longitudinal  Cross-sectional  Combination |
|  | Data for the proposed analysis will go through March 2011. We plan to describe the study population using means, standard deviations, and percentages, with differences across key characteristics assessed using t-test, chi-squared and Kruskal-Wallis tests as appropriate. Mean/median unadjusted rates of hospitalization will be calculated and differences across subgroups will be assessed using a Poisson regression model with length of follow-up as an offset term to account for varying duration of follow-up. These will be generated overall and by all primary predictors and covariate levels from baseline. Mean/median length of stay and unadjusted in-hospital death rates per 100 hospital stays will be calculated overall and by all proposed primary predictors and covariates using values from the closest study visit within a year prior to the hospitalization. The non-independence of observations will be addressed using robust variance estimation.  The Agency for Healthcare Research and Quality has released a categorization method to group ICD-9 diagnosis codes into diagnosis categories known as the Clinical Classifications Software (CCS)(16). We plan to apply the CCS classifications to the primary ICD-9 admitting diagnosis code for each hospitalization to characterize the type of hospitalization. We will then calculate the rates of each of these categories of hospitalization overall and by level of eGFR, the presence of proteinuria, age, and diabetes status. An Appendix table will list all observed primary codes for hospitalizations within the CRIC cohort. |
| **Relationship of the Proposed Manuscript to other CRIC Abstracts, Manuscripts, and Approved/Pending Manuscript Proposals** (See list of approved manuscripts at [www.cristudy.org](http://www.cristudy.org)) | This is the first CRIC manuscript proposal examining the burden of hospitalizations in our participant population. |
| **Proposed Mock-Up Tables and Figures** | See below |
| **References** | 1. Coresh J, Selvin E, Stevens LA, et al. Prevalence of chronic kidney disease in the United States. JAMA, 2007. 298(17):2038-2047.  2. U.S. Renal Data System. USRDS 2012 Annual Data Report: Atlas of Chronic Kidney Disease and End-Stage Renal Disease in the United States. Bethesda, MD: National Institutes of Health, National Institute of Diabetes and Digestive and Kidney Diseases; 2012.  3. Daratha KB, Short RA, Corbett CF, Ring ME, Alicic R, Choka R, et al. Risks of subsequent hospitalization and death in patients with kidney disease. Clin. J. Am. Soc. Nephrol. Cjasn. 2012 Mar;7(3):409–16.  4. Nitsch D, Nonyane BAS, Smeeth L, Bulpitt CJ, Roderick PJ, Fletcher A. CKD and hospitalization in the elderly: a community-based cohort study in the United Kingdom. Am. J. Kidney Dis. Off. J. Natl. Kidney Found. 2011 May;57(5):664–72.  5. Bello AK, Hemmelgarn B, Lloyd A, James MT, Manns BJ, Klarenbach S, et al. Associations among estimated glomerular filtration rate, proteinuria, and adverse cardiovascular outcomes. Clin. J. Am. Soc. Nephrol. Cjasn. 2011 Jun;6(6):1418–26.  6. Meng L, Ding W, Shi L, Jiang J, Liu Z, Gong Y. [Cardiovascular events in patients with chronic kidney disease]. Zhonghua Xin Xue Guan Bing Za Zhi. 2009 Jan;37(1):53–5.  7. Collins AJ, Li S, Gilbertson DT, Liu J, Chen S-C, Herzog CA. Chronic kidney disease and cardiovascular disease in the Medicare population. Kidney Int. Suppl. 2003 Nov;(87):S24–31.  8. Go AS, Chertow GM, Fan D, McCulloch CE, Hsu C. Chronic kidney disease and the risks of death, cardiovascular events, and hospitalization. N. Engl. J. Med. 2004 Sep 23;351(13):1296–305.  9. Hamaguchi S, Tsuchihashi-Makaya M, Kinugawa S, Yokota T, Ide T, Takeshita A, et al. Chronic kidney disease as an independent risk for long-term adverse outcomes in patients hospitalized with heart failure in Japan. Report from the Japanese Cardiac Registry of Heart Failure in Cardiology (JCARE-CARD). Circ. J. Off. J. Jpn. Circ. Soc. 2009 Aug;73(8):1442–7.  10. Dalrymple LS, Katz R, Kestenbaum B, de Boer IH, Fried L, Sarnak MJ, et al. The risk of infection-related hospitalization with decreased kidney function. Am. J. Kidney Dis. Off. J. Natl. Kidney Found. 2012 Mar;59(3):356–63.  11. Sood P, Kumar G, Nanchal R, Sakhuja A, Ahmad S, Ali M, et al. Chronic kidney disease and end-stage renal disease predict higher risk of mortality in patients with primary upper gastrointestinal bleeding. Am. J. Nephrol. 2012;35(3):216–24.  12. Khan SS, Kazmi WH, Abichandani R, Tighiouart H, Pereira BJG, Kausz AT. Health care utilization among patients with chronic kidney disease. Kidney Int. 2002 Jul;62(1):229–36.  13. Mix T-CH, St peter WL, Ebben J, Xue J, Pereira BJG, Kausz AT, et al. Hospitalization during advancing chronic kidney disease. Am. J. Kidney Dis. Off. J. Natl. Kidney Found. 2003 Nov;42(5):972–81.  14. Feldman HI, Appel LJ, Chertow GM, Cifelli D, Cizman B, Daugirdas J, et al. The Chronic Renal Insufficiency Cohort (CRIC) Study: Design and Methods. J. Am. Soc. Nephrol. Jasn. 2003 Jul;14(7 Suppl 2):S148–153.  15. Lash JP, Go AS, Appel LJ, He J, Ojo A, Rahman M, et al. Chronic Renal Insufficiency Cohort (CRIC) Study: baseline characteristics and associations with kidney function. Clin. J. Am. Soc. Nephrol. Cjasn. 2009 Aug;4(8):1302–11.  16. Agency for Healthcare Research and Quality. HCUP CCS Fact Sheet. Healthc. Cost Util. Proj. Hcup [Internet]. 2012 Jan [cited 2013 Jun 29]; Available from: www.hcup-us.ahrq.gov/toolssoftware/ccs/ccsfactsheet.jsp |

Changes to Original Analytical Plan

1. Prior to original submission
   1. Extended follow up time from 2011 to 2018 due to availability of adjudicated hospitalization data in the CRIC Study
   2. Added the National Inpatient Sample to serve as an illustrative comparator to the CRIC Study
   3. Investigated the interaction of eGFR and proteinuria on hospitalization rates
   4. Decided to not include duration of hospitalization stay in order to keep the current (and first) paper describing hospitalizations in CRIC more concise and easier to interpret
2. In response to PLOS Medicine review
   1. Investigate the association of age, systolic blood pressure, urine protein-to-creatine ratio and eGFR as modelled continuously with each type of hospitalization rate
