# Supplementary material for: Hospitalizations among adults with chronic kidney disease in the United States: A cohort study
Source: PLoS Med. 2020 Dec 11;17(12):e1003470. doi: 10.1371/journal.pmed.1003470 (PMC7732055; doi:10.1371/journal.pmed.1003470)
Supplement: Attachment — Submitted filename: PLOS_Medicine_Response_to_Reviewers.docx [file pmed.1003470.s015.docx]

**Reviewer 1:
I confine my remarks to statistical aspects of this paper. The general approach is fine, but I have a couple issues to resolve before I can recommend publication.**

1. **The biggest one is that continuous independent variables should not be categorized. In *Regression Modeling Strategies* Frank Harrell lists 11 problems that this causes and sums up "nothing could be more disastrous". I wrote a blog post taking a graphical look at what can happen:** [**https://medium.com/@peterflom/what-happens-when-we-categorize-an-independent-variable-in-regression-77d4c5862b6c?source=friends_link&sk=1428cd15968e218268121dc507ce8025**](https://medium.com/@peterflom/what-happens-when-we-categorize-an-independent-variable-in-regression-77d4c5862b6c?source=friends_link&sk=1428cd15968e218268121dc507ce8025)

Thank you for this suggestion. We have re-run the analyses with the independent variables modelled as continuous and have graphed the model-based curves, which depict a linear relationship between each of the continuous variables and rates of hospitalization (displayed below). Since each of the variables was observed to have a linear relationship with hospitalization rates, we prefer to report the results of the original analyses because the categorical variables represent clinically meaningful cut points and the results are easier to communicate to a clinical audience. We have included the graphs below as S1 Fig.

| **Age (year)** | | |
| --- | --- | --- |
| All Hospitalizations | Cardiovascular Hospitalizations | Non-Cardiovascular Hospitalizations |
|  |  |  |
| **Estimated Glomerular Filtration Rate (eGFR, ml/min/1.73m^2^)** | | |
| All Hospitalizations | Cardiovascular Hospitalizations | Non-Cardiovascular Hospitalizations |
|  |  |  |
| **Urine Protein-to-Creatinine Ratio (UPCR, mg/g)** | | |
| All Hospitalizations | Cardiovascular Hospitalizations | Non-Cardiovascular Hospitalizations |
|  |  |  |
| **Systolic Blood Pressure (SBP, mmHg)** | | |
| All Hospitalizations | Cardiovascular Hospitalizations | Non-Cardiovascular Hospitalizations |
|  |  |  |

2. **Other issues:  p. 7 Please operationalize all variables.**

Thank you for noting our lack of detail. We have included this covariate information in the statistical analysis section (page 9).

3. **p 9 Give the SD for age**

We apologize for not including the SD for age. It has now been included in Table 1.

4. **Fig 1: Use a line plot for age and do not use "dynamite plots" (see e.g** [**http://biostat.mc.vanderbilt.edu/wiki/Main/DynamitePlots**](http://biostat.mc.vanderbilt.edu/wiki/Main/DynamitePlots)**)**

Thank you for this suggestion and the reference. As per the response to #1, we prefer to present the results of the original analyses for the purposes of clinical interpretability. We have included the graph of age and rate of hospitalization when age was modelled continuously as an appendix. Please see S1 Fig.

5. **Fig 3: Do not use 3 dimensional plots.  William S. Cleveland has shown that these cannot be accurately read. But the variables should be continuous, so this plot will change a lot.**

Thank you for bringing the limitations of 3-D plots to our attention. We have created a new version of Figure 3 to avoid these limitations. Please also see response to comment #1 to discuss modelling continuous variables.

**Reviewer 2:
Schrauben et al. explored hospitalization rates among persons with CKD in the CRIC observational study and contrasted with hospitalization rates in the Nationwide Inpatient Sample (NIS) for 2012. They found higher hospitalization rate in the CRIS than NIS, predominantly related to diverse illnesses other than cardiovascular disease.**

**I offer the following suggestions for consideration:**

1. **The concluding statement in the abstract "Adults with CKD appear to have a substantially higher hospitalization rate than those without CKD" is not supported by the results.**

Thank you for your comment. The conclusion has been revised to read:

“In this study, we observed that adults with CKD have a higher hospitalization rate than the general population that is hospitalized, and even moderate reductions in kidney function were associated with elevated rates of hospitalization. Causes of hospitalization were predominantly related to cardiovascular disease, but other causes contributed, particularly, genitourinary, digestive, and endocrine, nutritional and metabolic illnesses. High levels of proteinuria were observed to have the largest association with hospitalizations across a wide range of kidney function levels.”

2. **On page 8, under statistical analysis please specify the start of follow-up in the CRIC cohort and the median follow-up time. Also, describe how the hospitalization rates were calculated for the NIS.**

Thank you for bringing the lack of detail to our attention. We have now specified that the follow up time in CRIC was 2003 to 2018 with a median follow up time of 9.6 years (Results, page 10). In addition, the following text was added to provide more detail on how the NIS hospitalization rates were calculated (statistical analysis section, page 10):

Hospitalization rates in the NIS were calculated by first applying discharge weights to the NIS discharge data to estimate the number of hospital discharges that occurred in the US in 2012, then divided this number by the US population determined by the census.

3. **Please indicate the reasoning for selecting the year 2012 for NIS data.**

We have included the rationale for selecting 2012 for NIS data in the manuscript and it reads:

“The year 2012 was meant to be an illustrative comparison to the CRIC cohort and was chosen because it represented a year when the entire original CRIC cohort was enrolled and hospitalization ascertainment was optimized.”

4. **NIS data represent the number of discharge diagnoses per hospital stay, not per patient, so the incidence of any hospitalization is overestimated, an aspect that should be discussed as a limitation. Along this line, it is not clear if the numerator for the CRIC data included the first (incident) hospitalization during the follow-up period, or any hospitalizations. If the former is true, the authors may consider discussing how this aspect affects the comparison with NIS rates. The authors ought to specify how the numerator was counted in each of the two data sources.**

For each hospitalization in the NIS data, we only chose the primary discharge diagnosis and did not include of all of the diagnoses associated with the hospitalization. The hospitalizations in CRIC were also determined in the same manner to avoid overestimation. In CRIC, the total number of hospitalizations that occurred during the follow up period was accounted for. In both data sets, the rate was ascertained by summing hospitalizations for a given age group over the person-time of the follow-up in that group. We have now included this information in the statistical analysis section (page 10). We also have included more information regarding how the hospitalization rates in NIS were calculated – as discussed in point 2 above.

**Reviewer 3:**

**This is an interesting paper and highlights a major issue for patients with even fairly mild chronic kidney disease. The data presented genuinely contribute to novel understanding of this issue and in my opinion will be useful for research, healthcare policy and resource allocation. The CRIC cohort is well known, but this looks like a completely new and more detailed analysis compared to any of their other work. To my reading, this is well presented, the analyses look sound and the tables and figures are informative. I have little to suggest to this and I think this could be a very useful paper for others working in this field.**

**My main comments are around the grammar, or messaging in the paper**

1. **Abstract.
   I presume they mean 500mg/g of proteinuria? - there is no explanation as to what this measurement refers to. Also why present this threshold in the abstract when most of the thresholds for uPCR in the paper are 150mg/g. Is this correct? I presume this is as 500mg/g is the upper category - it could be clarified as to why this value appears where it does.**

We appreciate you noting our lack of detail regarding proteinuria. We have now updated the abstract to clarify these details.

**Although I accept that the majority of illnesses are not cardiovascular, the single biggest disease by far affecting these patients is cardiovascular disease as their analysis shows (31.8% compared to 8.7% for the next condition down). I think the message should be slightly rephrased to make sure that the message about CVD is not diminished too much.**

Thank you for your comment. We agree that we might have diminished the role of cardiovascular disease hospitalizations and have rephrased our messaging in the concluding statements. Please see conclusion in abstract.

It now reads: “In this study, we observed that adults with CKD have a higher hospitalization rate than the general population that is hospitalized, and even moderate reductions in kidney function were associated with elevated rates of hospitalization. Causes of hospitalization were predominantly related to cardiovascular disease, but other causes contributed, particularly, genitourinary, digestive, and endocrine, nutritional and metabolic illnesses. High levels of proteinuria were observed to have the largest association with hospitalizations across a wide range of kidney function levels.”

2. **Main text
   They should go through this carefully for units etc. I found a few instances where the units had dropped off inappropriately e.g. for GFR on p10. There are probably others. I don't view it as the role of reviewer to be a proof reader!**

Thank you for noting our errors. We apologize for overlooking these errors and thank you for bringing them to our attention. We have thoroughly reviewed the manuscript and corrected any missing units or grammatical errors when found.

**Editorial Comments:**

**Please revise your title according to PLOS Medicine's style. Your title must be nondeclarative and not a question. It should begin with main concept if possible. "Effect of" should be used only if causality can be inferred, i.e., for an RCT. Please place the study design ("A randomized controlled trial," "A retrospective study," "A modelling study," etc.) in the subtitle (ie, after a colon).**

The title has been revised to “Description of Hospitalizations Among Adults with Chronic Kidney Disease: An Observational Longitudinal Cohort Study”.

**Please structure your abstract using the PLOS Medicine headings (Background, Methods and Findings, Conclusions). Please combine the Methods and Findings sections into one section, “Methods and findings”.**

The abstract has been re-structured to the PLOS Medicine style.

**Abstract Methods and Findings:**

- **Please ensure that all numbers presented in the abstract are present and identical to numbers presented in the main manuscript text.**
- **Please include the study design, population and setting, number of participants, years during which the study took place, length of follow up, and main outcome measures.**
- **Please quantify the main results (with 95% CIs and p values).**
- **Please include the important dependent variables that are adjusted for in the analyses.**

The abstract methods and findings have been updated to reflect these suggested changes.

**Abstract Conclusions:**

- **Please address the study implications without overreaching what can be concluded from the data; the phrase "In this study, we observed ..." may be useful.**
- **Please interpret the study based on the results presented in the abstract, emphasizing what is new without overstating your conclusions.**
- **Please avoid vague statements such as "these results have major implications for policy/clinical care". Mention only specific implications substantiated by the results.**
- **Please avoid assertions of primacy ("We report for the first time....")**

The abstract conclusions have been updated to reflect the suggested changes.

**Methods- please briefly state where the participants were recruited in the US.**

The methods now include this information.

**On page 8- please remove the information regarding the funding source as this is pulled from the article meta-data only.**

The funding information has been removed.

**Please provide 95% confidence intervals and p values throughout as needed. Please note we need exact p values unless p<0.001.**

The 95% confidence intervals and p-values have been provided when necessary throughout the manuscript.

**Did your study have a prospective protocol or analysis plan? Please state this (either way) early in the Methods section.**

**a) If a prospective analysis plan (from your funding proposal, IRB or other ethics committee submission, study protocol, or other planning document written before analyzing the data) was used in designing the study, please include the relevant prospectively written document with your revised manuscript as a Supporting Information file to be published alongside your study, and cite it in the Methods section. A legend for this file should be included at the end of your manuscript.
b) If no such document exists, please make sure that the Methods section transparently describes when analyses were planned, and when/why any data-driven changes to analyses took place.
c) In either case, changes in the analysis-- including those made in response to peer review comments-- should be identified as such in the Methods section of the paper, with rationale.**

The original planning document and changes to the analytical plan are included as Supporting Information (S2 Text) and referenced in the Methods section, as well as responses to peer review.

**Please present and organize the Discussion as follows: a short, clear summary of the article's findings; what the study adds to existing research and where and why the results may differ from previous research; strengths and limitations of the study; implications and next steps for research, clinical practice, and/or public policy; one-paragraph conclusion.**

The organization of the discussion is provided in the suggested format.  **STROBE- please specify where individual items on the checklist can be found using paragraphs and sections.**

The STROBE statement has been updated with the requested information (S1 Table STROBE Checklist).

**The Data Availability Statement (DAS) requires revision. For each data source used in your study:

a) If the data are freely or publicly available, note this and state the location of the data: within the paper, in Supporting Information files, or in a public repository (include the DOI or accession number).
b) If the data are owned by a third party but freely available upon request, please note this and state the owner of the data set and contact information for data requests (web or email address). Note that a study author cannot be the contact person for the data.
c) If the data are not freely available, please describe briefly the ethical, legal, or contractual restriction that prevents you from sharing it. Please also include an appropriate contact (web or email address) for inquiries (again, this cannot be a study author).**

The DAS has been revised. It now reads:

The data from the CRIC Study that support the findings of this study are available upon request at the NIDDK Repository at https://repository.niddk.nih.gov/studies/cric/

You must be a registered user on the NIDDK Repository website to access and use the data and sample request systems. You can register at https://repository.niddk.nih.gov/register/

Instructions for how to make a request are found at https://repository.niddk.nih.gov/pages/overall_instructions/

Please contact the NIDDK Repository Communications team ([niddk-cr@imsweb.com](mailto:niddk-cr@imsweb.com)) should you have any questions about the requests or are unsure of which one fits your needs.

The data from the National Inpatient Sample (NIS) that support the findings of this study are available upon request and purchase from the Healthcare Cost and Utilization Project (HCUP). Restrictions apply to the availability of these data, which were used under license for this study. Data are available for purchase online through the Online HCUP Central Distribtuion with permission of HCUP at: <https://www.hcup-us.ahrq.gov/tech_assist/centdist.jsp>

Questions about purchasing databases can be directed to the HCUP Central Distributor:

Email: [HCUPDistributor@AHRQ.gov](mailto:HCUPDistributor@AHRQ.gov) 
Telephone: (866) 556-4287 (toll free) 
Fax: (866) 792-5313 (toll free)
