# Supplementary material for: Hospitalizations among adults with chronic kidney disease in the United States: A cohort study
Source: PLoS Med. 2020 Dec 11;17(12):e1003470. doi: 10.1371/journal.pmed.1003470 (PMC7732055; doi:10.1371/journal.pmed.1003470)
Supplement: Attachment — Submitted filename: Response to Reviewers.docx [file pmed.1003470.s016.docx]

**Requests from Editors:**

**Title- I suggest revising to “ Hospitalizations Among Adults with Chronic Kidney Disease in the United States: A Cohort Study" or similar.**

Thank you, the title has now been revised to this suggestion.

**Abstract:**

- **Please provide brief participant demographics and where the cohort is located**
- **Please include 2-3 limitations of your study in the last sentence of the methods and findings section**
- **Please revise to ""had" in the first line of the conclusions**

The requested information and revision have now been included in the revised abstract.

**Throughout- please place reference brackets before punctuation, providing a space in between.**

**Please provide p-values up to three decimal places throughout.
Page 16 first paragraph third sentence contains an additional “to”**

We have now revised the manuscript to reflect these suggestions.

**STROBE checklist- please remove page numbers as these are likely to change during publication. Please use paragraphs and sections instead. The STROBE and protocol documents can be uploaded as separate attachments, referred to in the methods**

The STROBE checklist has been updated and uploaded as a separate attachment.

**Please provide access details for ref 19, 36. Reference 1 needs 6 author names (+ page numbers?)**

The references have been edited to include the requested missing information.

**At the end of the ms, the acknowledgements are mainly funding, I think, which is in the submission form and can be removed.**

This information has been removed.

**Please ensure all p-values provided are up to three decimal places. All iterations of p<0.0001 should be changed to p<0.001**

We have updated all p-values to include three decimal places.


**Comments from Reviewers:**

**Reviewer #1: The authors have addressed my concerns and I now recommend publication**

Peter Flom

Thank you so much for your thoughtful review.

**Reviewer #2: The authors' responses are satisfactory and i have no further comments. Thank you.**

We thank you for your time in reviewing this manuscript.


**Reviewer #3: I have no further comments**

Thank you for your time and feedback.
